# The Conventions at Old Point Comfort

**Published:** 1900-08

**Authors:** 


### THE CONVENTIONS AT OLD POINT COMFORT.

The National Dental Association convened at the above place on Tuesday, July 10, 1900. The attendance was larger than anticipated, and quite filled the large meeting-room in the Hotel Chamberlin. This room was a great improvement over that of the preceding year at Niagara Falls.

The country, even as far as the Pacific Coast, was well represented, several delegates from the latter section being drawn there in attendance upon the National Association of Dental Faculties, thus demonstrating the value of holding the meetings about the same time and place, insuring co-operation in interest and work.

The National Association was called to order at the appointed time, by the President, Dr. B. Holly Smith, of Baltimore.

The good results of having an "Executive Council" to consider routine business was fully demonstrated, and the Association was able to begin at once with the real work of the body, reading of papers and discussions.

The President's address was an able presentation of many subjects connected with dental education and the management of the Association, especially in the necessity for a reorganization of the sections. In the final report of the committee appointed to consider this address it was recommended that a special committee take this portion into consideration and, if possible, devise a plan whereby greater vitality could be infused into these branches of the organization. This committee subsequently reported, creating a sub-committee from each section, termed a "commission," whose duty it would be to have conducted original investigations, keep in full critical touch with the dental literature of the year, and report upon the work to the section, and was given power to draw upon the general treasury to pay the expense of original work. The sections reorganized under this plan, and it is hoped that the result will be to encourage investigation and, at the same time, eliminate amateurish productions and a constant repetition of ideas previously worked out.

The evening session of Tuesday was mainly occupied in the consideration of a paper by Dr. C. N. Johnson, of Chicago, on "Inlays: Their Advantages and Limitations." The general trend of the paper was a surprise to many, as it was a complete reversal of the opinions expressed at the meeting last year. The essayist was opposed to the indiscriminate use of inlays for various reasons, —difficulty in making them permanent, variation in color, etc. The support which this paper received from those who formerly strongly advocated the use of inlays indicates that the general thought is now tending towards limiting their use. While the methods of preparation may be defective, it is to be hoped that these difficulties may eventually be overcome, for they certainly have a value from an æsthetic point of view not equalled by any other of the recognized operations.

Dr. Ames advocated gold inlays, and this received commendation by some for large cavities in posterior teeth.

The principal papers in subsequent sessions were "A New Cavity Preparation for Distal Cavities," by Dr. E. R. Wedelstaedt, of St. Paul; "Art in Prosthetic Dentistry," by Dr. Mary E. Gallup, Boston; "A System of Removable Bridge-Work," by Dr. W. E. Griswold, Denver; "Antiseptic Surgery of the Face and Mouth," by Dr. W. H. G. Logan, Chicago; "The Evolution of the Bunodont from the Haplodont Forms of Teeth," by Dr. A. H. Thompson, Topeka, Kan. Several additional papers were read of more or less value on ethics and general dental education, but it must be acknowledged that, with two or three exceptions, there was a marked falling off in scientific interest from the papers of the previous year. This was not disappointing, as it was anticipated this would be the result of the excess of effort in this direction at Niagara Falls. It is expected that the reorganization of the sections, on the plan mentioned, will do away with this unstable character in the work of the Association.

While the meetings were pleasant and the discussions generally intelligently confined to the subject-matter of the papers, it must be stated that the proceedings lacked interest, and that those who came expecting to return laden with ideas doubtless left disappointed. Such meetings, however, in the opinion of the writer, are never a failure. If they subserve no other purpose, they at least infuse new motive force into professional life, and those who attend must go away feeling its revivifying influence.

The meeting elected Dr. G. V. Black, Chicago, President for the ensuing year, and selected Milwaukee as the place of meeting, the first Tuesday in August, 1901.

The Association of Dental Faculties met July 13, 1900, at the Hygeia Hotel, Old Point Comfort. The work of this body was conducted with more harmony than at previous meetings. The elimination of the vexatious problems connected with State and national examining boards had largely to do with this, the business being mainly confined to educational subjects and the admission and control of colleges.

There was only one unpleasant feature connected with its deliberations, and that was the introduction of a lawyer to defend a college charged with not living up to the standard of this Association. This was exceedingly annoying, and really placed the college in a very unfavorable light before the other members. This should never be tolerated again, and a college so offending should be placed outside the pale of professional recognition.

The Association was not prepared to make any advance either in time or the curriculi of colleges, and therefore the situation continues practically the same as it was previous to this meeting. It remains now for the larger schools to take the initiative and declare for a four years' course of nine months each, and this we feel assured will be done, not alone for the good of the student, but to enable faculties to complete the work of training undergraduates with the least possible friction and with the best results.

The National Association of Dental Examiners met at the same time and place, but the writer is not aware of any special legislation affecting colleges. The harmony between the two Associations was not disturbed in any way. The nearest approach to this was an introduction of a resolution in the Faculties to do away with the endorsement of State boards on certificates of application for membership in this Association. This has always been required, and has been a constant source of trouble. When it was adopted by the National Association of Dental Faculties, it was essential to a proper understanding of the position occupied by the colleges in the State, but now, with the facilities for the personal examination required, the method is antiquated and a constant source of friction. The Association was nearly equally divided on the question, but the rule still remains among the laws for the ensuing year, and it is hoped for a not much longer period. Why some men seem eager to do the work of the State boards appointed by the governors is a problem, but that many constantly truckle to these bodies is an assured but a melancholy fact. The National Association of Dental Faculties can have nothing to do legitimately with any such organizations, however worthily they may be constituted. The peace of the present is simply a truce. This may not be broken, but there are latent forces at work which sooner or later will cause violent disturbance. It is useless to expect any other result; in fact, such a peace is not desirable so long as these boards are composed, as they generally are, of political favorites and with little or no regard to the ability of the men composing them. Ordinarily they are unequal to traversing intelligently the work of the colleges. The exceptions to this are so few that they need not be considered.

This year, so far as dental conventions are concerned, may be regarded as a weak year. This must be expected to follow in regular course. It is anticipated that 1901 will show a marked improvement. There is certainly room for it, and the censorship of the sections in the National Association must be used unsparingly. This meeting was rendered exceedingly tedious at times by papers of tiresome length and repetition of ideas. Essayists should be limited to time, and when this is exceeded the gavel of the presiding officer should descend without fear or favor.

